# Effect of Baseline Values of Renal Prognosis-related Factors on Their Changes after Initiating Tofogliflozin Treatment: A Retrospective Study in Japanese Patients with Type 2 Diabetes and Renal Impairment

**DOI:** 10.31662/jmaj.2024-0128

**Published:** 2024-09-06

**Authors:** Suzuko Matsumoto, Hiroyuki Ito, Hideyuki Inoue, Chiaki I, Shun Miura, Shinichi Antoku, Tomoko Yamasaki, Toshiko Mori, Michiko Togane

**Affiliations:** 1 Department of Diabetes, Metabolism and Kidney Disease, Edogawa Hospital, Tokyo, Japan

**Keywords:** Sodium-glucose cotransporter 2 inhibitors, hematocrit, hemoglobin, tofogliflozin; uric acid, diabetic kidney disease

## Abstract

**Introduction:**

This study investigated the relationships between changes in renal prognosis-related factors after initiating tofogliflozin and the corresponding baseline values in clinical practice in Japanese patients with type 2 diabetes.

**Methods:**

We investigated the relationships between changes in hematocrit, hemoglobin, systolic blood pressure (sBP), urinary protein excretion (uPE), serum uric acid (sUA), and estimated glomerular filtration rate (eGFR) 12 months after initiating tofogliflozin (20 mg) and their corresponding baseline values in 130 patients with type 2 diabetes. The subjects were divided into two groups: normal (≥60 mL/min/1.73 m^2^, n = 87) and low (<60 mL/min/1.73 m^2^, n = 43) eGFR.

**Results:**

Although the change in eGFR was negatively correlated with the baseline value in the normal-eGFR group, no significant correlation was found between the change in eGFR and baseline value in the low-eGFR group. Although changes in hematocrit (r = −0.39, P = 0.01) and hemoglobin (r = −0.36, P = 0.02) levels were significantly negatively correlated with corresponding baseline values in the low-eGFR group, no significant correlations were observed in the normal-eGFR group. Changes in sBP, uPE, and sUA were significantly negatively correlated with the corresponding baseline values in both the normal- and low-eGFR groups. None of the correlation coefficients between the normal- and low-eGFR groups showed a significant difference.

**Conclusions:**

Favorable changes in renal prognosis-related factors after tofogliflozin therapy may contribute to renoprotection in patients with type 2 diabetes and poor corresponding baseline values, despite the presence of renal impairment.

## Introduction

Sodium-glucose cotransporter 2 (SGLT2) inhibitors suppress the decline in estimated glomerular filtration rate (eGFR), even in patients with type 2 diabetes and renal impairment ^(1), (2)^. In addition to improving hyperglycemia through urinary glucose excretion, various underlying mechanisms have been identified for this renoprotective effect ^(3)^.

We also showed that administration of SGLT2 inhibitors preserved the eGFR with a reduction in blood pressure, urinary protein excretion (uPE), and serum uric acid (sUA) levels and elevation in hemoglobin level in patients with type 2 diabetes and an eGFR of <60 mL/min/1.73 m^2^ ^(4), (5)^. Changes in these factors are considered important for renal prognosis. According to a recently reported *post hoc* mediation analysis of the EMPA-REG OUTCOME trial, changes in hematocrit and hemoglobin levels were the strongest mediators of renal benefit after initiating empagliflozin in patients with type 2 diabetes and cardiovascular disease ^(6)^. In univariable analyses adjusted for the updated mean and baseline values of the potential mediators, changes in hematocrit, hemoglobin, sUA, urinary albumin-to-creatinine ratio (uACR), and systolic blood pressure (sBP) values mediated 99.5%, 79.4%, 33.2%, 31.0%, and 13.0% of the empagliflozin treatment effect on the composite kidney outcome, respectively. The authors indicated that an erythropoietic mechanism potentially linked to the alleviation of organ hypoxia played a crucial role in empagliflozin-induced renoprotection.

It remains unclear which baseline characteristics of patients exhibit favorable changes in the aforementioned mediators during SGLT2 inhibitor therapy. Thus, we investigated the relationships between changes in renal prognosis-related factors after initiating tofogliflozin and the corresponding baseline values in Japanese patients with type 2 diabetes, including those with renal impairment. The outcomes and exposures in this study were changes in renal prognosis-related factors and their baseline values, respectively. The results of this study are expected to clarify the clinical features of patients who are more likely to benefit from SGLT2 inhibitor therapy in real clinical practice.

## Materials and Methods

### Study subjects

We reanalyzed our previous retrospective study ^(5)^ to determine the effects of tofogliflozin on eGFR in Japanese patients with type 2 diabetes. In brief, among 250 patients with type 2 diabetes who were prescribed 20 mg of tofogliflozin once daily (Deberza^Ⓡ^ tablets; Kowa Company, Ltd., Nagoya, Japan) over 12 months at the Department of Diabetes, Metabolism and Kidney Disease from March 2019 to April 2021, 130 patients in the full analysis set (87 patients with an eGFR value of ≥60 mL/min/1.73 m^2^ [normal-eGFR group] and 43 patients with an eGFR value of <60 mL/min/1.73 m^2^ [low-eGFR group]) were included in this study.

This study excluded individuals who started iron preparation or glucagon-like peptide-1 receptor agonist treatment during the observation period and those whose antihypertensive agents, diuretics, or urate-lowering agent therapies were changed during the observation period.

The primary outcomes were the relationships between changes in hematocrit and hemoglobin levels during the 12-month follow-up of initiating tofogliflozin and their corresponding baseline values in the normal- and low-eGFR groups. The relationships between changes in sBP, uPE, sUA, HbA1c, and eGFR and their corresponding baseline values were also examined as secondary outcomes.

### Measurements

The uPE was determined using urine test strips (Uriflet S; ARKRAY, Inc., Kyoto, Japan) and an automatic analyzer (Austin MAX AX 4280; ARKRAY, Inc.) in a random spot urine test. Proteinuria was classified into five levels: (−), (±), (1+), (2+), and (3+), which correspond to 0, 15, 50, 150, and 325 mg/dL, respectively ^(4), (5), (7)^.

### Statistical analyses

All data are presented as the mean ± standard deviation. The χ^2^ test was used for between-group comparisons of categorical variables. Wilcoxon’s signed-rank test was used to determine the significance of differences in continuous variables. Wilcoxon’s rank sum test was used to assess the significance of differences in hematocrit, hemoglobin, sBP, uPE, sUA, HbA1c, and eGFR during the observation period compared with the corresponding baseline values. The normal distribution of continuous variables was tested using the Shapiro-Wilk normality test. Pearson’s correlation coefficient (r) was used to determine the relationship between two variables that showed a normal distribution, and Spearman’s correlation coefficient (r_s_) was used to determine the relationship between variables that did not show a normal distribution. Correlation coefficients were compared after Fisher’s z-transformation between the normal- and low-eGFR groups.

Statistical significance was set at P < 0.05 (two-tailed). JMP version 12.2.0 (SAS Institute, Cary, NC, USA) and Microsoft Excel were used to perform all statistical analyses.

## Results

### Changes in eGFR and renal prognosis-related factors

Table 1 shows the clinical characteristics of the study participants at baseline. Supplementary Appendix 1 presents the changes in renal prognosis-related factors from baseline to 12 months after initiating tofogliflozin. Hematocrit and hemoglobin levels were significantly increased in both the normal- and low-eGFR groups. The sBP, uPE, and HbA1c levels were significantly decreased in both the normal- and low-eGFR groups. Although sUA levels were significantly decreased in all participants, the reduction was not significant in the low-eGFR group. The eGFR was preserved in both the normal- and low-eGFR groups. The diastolic blood pressure and serum potassium levels did not significantly change.

**Table 1.** Clinical Background of the Study Population at Baseline.

			Groups divided by eGFR at baseline		
	N^†^	All subjects	Normal eGFR	Low eGFR	P
		(n = 130)	(n = 87)	(n = 43)	
Male subjects (%)	130	72	71	74	0.71
Age (years)	130	64 ± 12	61 ± 12	70 ± 10	<0.01
Duration of diabetes (years)	120	13 ± 9	11 ± 8	15 ± 10	0.03
Body weight (kg)	124	74.0 ± 14.2	74.0 ± 14.8	73.9 ± 13.1	0.85
BMI (kg/m^2^)	124	27.6 ± 4.8	27.6 ± 5.1	27.7 ± 4.4	0.78
Obesity (%)^††^	124	69	66	74	0.37
sBP (mmHg)	122	135 ± 15	134 ± 12	139 ± 18	0.23
dBP (mmHg)	119	80 ± 13	80 ± 13	79 ± 16	0.44
uPE (mg/dL)	121	36 ± 77	21 ± 49	66 ± 109	<0.01
Hematocrit (%)	130	42.7 ± 4.1	42.6 ± 3.8	43.0 ± 4.6	0.63
Hemoglobin (g/L)	130	143 ± 15	144 ± 14	142 ± 15	0.62
sUA (μmol/L)	118	305 ± 70	297 ± 71	319 ± 67	0.12
Serum potassium (mEq/L)	129	4.4 ± 0.4	4.4 ± 0.4	4.5 ± 0.4	0.05
HbA1c (%)	129	8.4 ± 1.5	8.4 ± 1.6	8.3 1.4	0.83
HbA1c (mmol/mol)	129	67.9 ± 16.5	68.2 ± 17.0	67.1 ± 15.5	0.83
eGFR (mL/min/1.73 m^2^)	130	68.9 ± 21.8	80.7 ± 15.3	44.6 ± 10.4	<0.01
Minimum, maximum		23.1, 141.8	60.3, 141.8	23.1, 59.9
75th percentile, median, 25th percentile		80.8, 70.0, 52.3	85.9, 77.3, 69.9	52.4, 46.2, 38.3

BMI, body mass index; sBP, systolic blood pressure; dBP, diastolic blood pressure; uPE, urinary protein excretion; sUA, serum uric acid; eGFR, estimated glomerular filtration rate. ^†^Estimated number; ^††^BMI of ≥25.0 kg/m^2^.

The change in HbA1c was significantly negatively correlated with the corresponding baseline values in both the normal- and low-eGFR groups (Supplementary Appendix 2A and 2B). Although the change in eGFR tended to be negatively correlated with the baseline value in the normal-eGFR group, no significant correlation was observed between the change in eGFR and the baseline value (Supplementary Appendix 2C and 2D).

### Relationships between renal prognosis-related changes and corresponding baseline values

**Figure 1.**
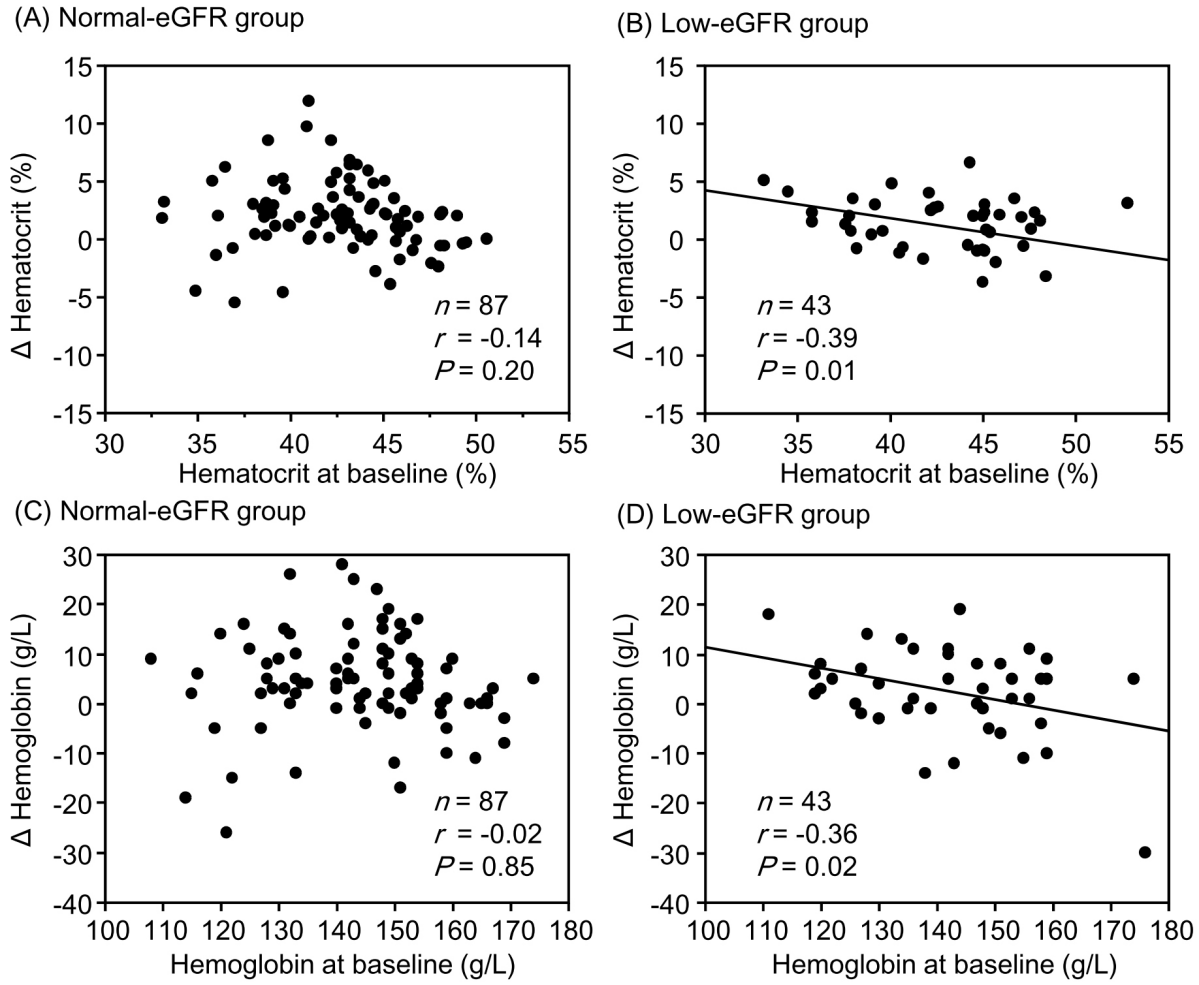
Relationship between changes in hematocrit and hemoglobin and their corresponding baseline values. Hematocrit levels in the normal- (A) and low-estimated glomerular filtration rate (eGFR) (B) groups. Hemoglobin levels in the normal- (C) and low-eGFR (D) groups.

**Figure 2.**
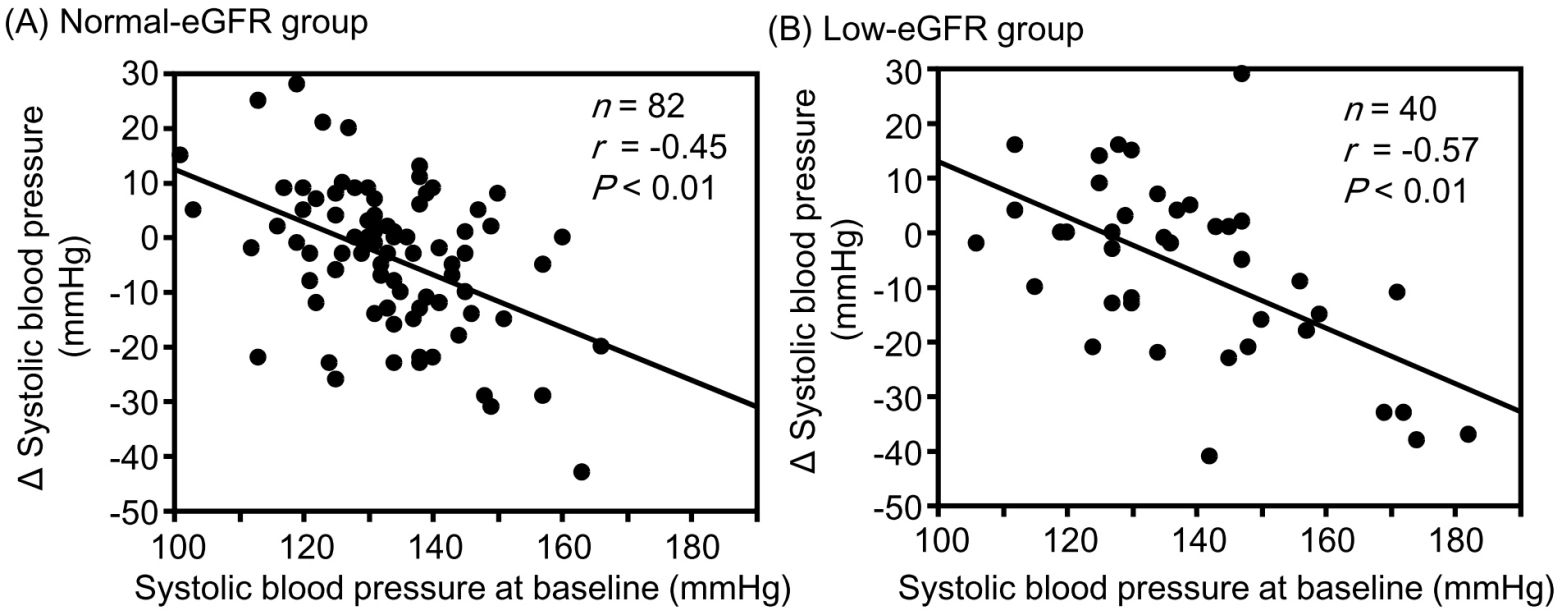
Relationship between changes in systolic blood pressure and baseline values. The normal- (A) and low-estimated glomerular filtration rate (eGFR) (B) groups.

**Figure 3.**
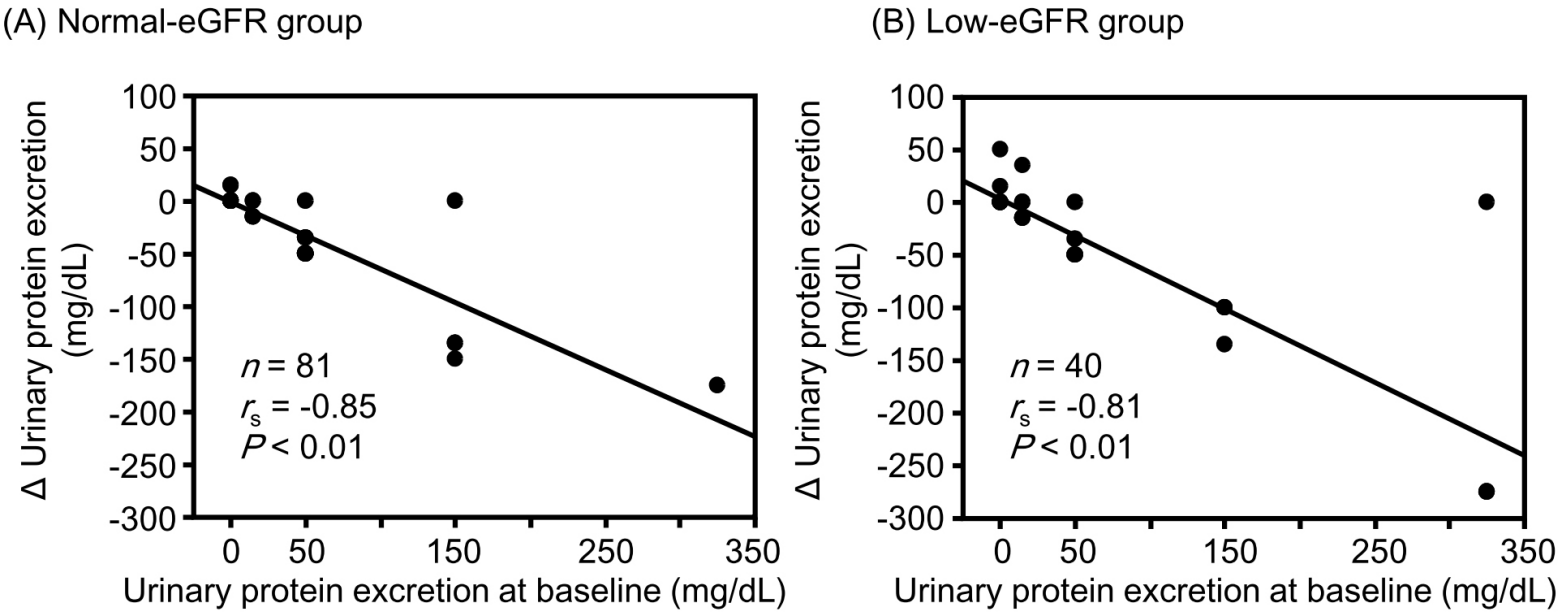
Relationship between changes in urinary protein excretion and the baseline values. The normal- (A) and low-estimated glomerular filtration rate (eGFR) (B) groups.

**Figure 4.**
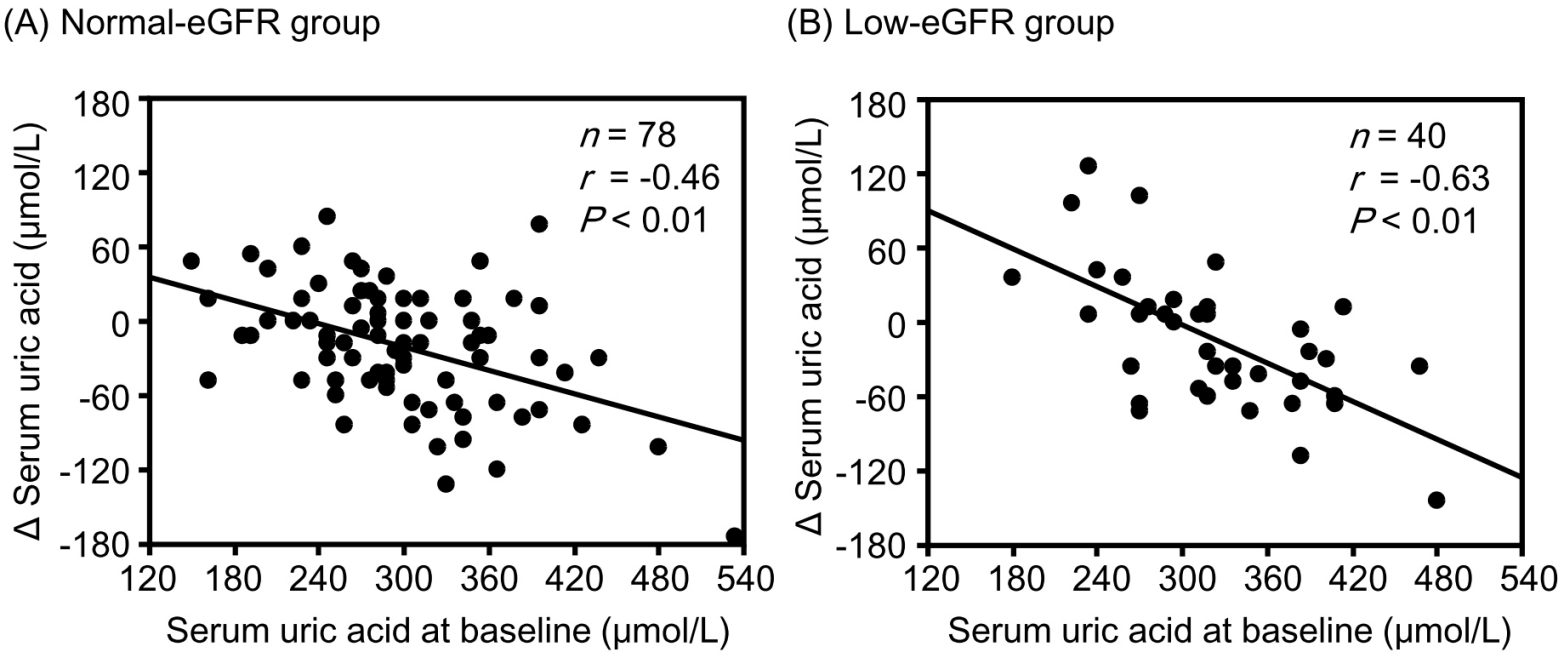
Relationship between serum uric acid and baseline values. The normal- (A) and low-eGFR (B) groups.

Although changes in hematocrit and hemoglobin levels were significantly negatively correlated with the corresponding baseline values in the low-eGFR group, no significant correlations were observed between them in the normal-eGFR group (Figure 1). The change in sBP was significantly negatively correlated with the corresponding baseline values in both the normal- and low-eGFR groups (Figure 2). The change in uPE (Figure 3) was also negatively correlated with the corresponding baseline values in the normal- and low-eGFR groups. The number of patients corresponding to the changes in uPE was 4 (−275 mg/dL), 1 (−175 mg/dL), 1 (−150 mg/dL), 3 (−135 mg/dL), 3 (−100 mg/dL), 11 (−50 mg/dL), 5 (−35 mg/dL), 11 (−15 mg/dL), 75 (0 mg/dL), 5 (15 mg/dL), 1 (35 mg/dL), and 1 (50 mg/dL). The change in sUA (Figure 4) was also negatively correlated with the corresponding baseline values in the normal- and low-eGFR groups.

### Correlation coefficients between the normal- and low-eGFR groups

Comparisons of the correlation coefficients between changes in hematocrit, hemoglobin, sBP, uPE, sUA, HbA1c, and eGFR and the corresponding baseline values between the normal- and low-eGFR groups are presented in Table 2. There were no significant differences in any of the correlation coefficients between the two groups. However, the correlation between the change in hemoglobin and the baseline value tended to be stronger in the low-eGFR group than in the normal-eGFR group. The correlation between changes in HbA1c levels and baseline values tended to be stronger in the normal-eGFR group than in the low-eGFR group.

**Table 2.** Correlation Coefficients between Changes in Renal Prognosis-Related Factors and Corresponding Baseline Values in the Normal and Low-eGFR Groups.

	Groups divided by eGFR at baseline				
	Normal-eGFR		Low-eGFR		P
	correlation coefficient	z	correlation coefficient	z	
Hematocrit	−0.14	−0.14	−0.39	−0.41	0.16
Hemoglobin	−0.02	−0.02	−0.36	−0.38	0.06
sBP	−0.45	−0.48	−0.57	−0.65	0.41
uPE	−0.85	−1.26	−0.81	−1.13	0.52
sUA	−0.46	−0.50	−0.63	−0.74	0.22
HbA1c	−0.77	−1.02	−0.57	−0.65	0.05
eGFR	−0.18	−0.18	0.02	0.02	0.29

sBP, systolic blood pressure; uPE, urinary protein excretion; sUA, serum uric acid; eGFR, estimated glomerular filtration rate.

## Discussion

In addition to hypertension and proteinuria, anemia ^(8), (9)^ and hyperuricemia ^(10), (11)^ are known to be associated with cardiovascular and renal events in patients with type 2 diabetes, although they are not in a serious condition. In this study, increases in hematocrit and hemoglobin and decreases in sBP, uPE, and sUA, which are closely associated with the renoprotective effect of SGLT2 inhibitors ^(3)^, were observed in both the normal- and low-eGFR groups. Because there were no changes in the drugs affecting these factors throughout the observation period, these fluctuations were likely caused by tofogliflozin therapy. If no intervention is provided to prevent deterioration, these factors often worsen over time in patients with renal impairment. Although the decrease in HbA1c was smaller in the low-eGFR group than in the normal-eGFR group, the favorable changes in these factors may be responsible for the preservation of eGFR in the low-eGFR group.

Among these factors, the most important for renoprotection were changes in hematocrit and hemoglobin, which were the primary outcomes in this study. Wanner et al. ^(6)^ reported that changes in hematocrit and hemoglobin were the strongest mediators, and those in sUA, uACR, and sBP contributed to the treatment effect of empagliflozin on composite kidney outcomes according to a mediation analysis of the EMPA-REG OUTCOME trial. These findings are consistent with the results of the mediation analysis of the CANVAS program ^(12)^. In this analysis, changes in red blood cell (RBC) count (56.7%), hematocrit (51.1%), hemoglobin (41.3%), sUA (35.4%), and sBP (8.9%) mediated the effect of canagliflozin on kidney outcomes. In a parsimonious multivariate model, erythrocyte concentration, sUA, and sBP maximized a cumulative mediation (115%), whereas the change in HbA1c was a negligible mediator at 0.5%. Furthermore, Doi et al. ^(13)^ reported that changes in hematocrit, hemoglobin, RBC count, and uACR at 52 weeks after initiating canagliflozin administration mediated significant risk reduction (47%, 41%, 40%, and 29%, respectively) according to a mediation analysis of the CREDENCE trial.

In a *post hoc* analysis of the EMPA-REG OUTCOME trial, a similar reduction in sBP and similar elevation in hemoglobin from baseline values between the empagliflozin and placebo groups were observed with an eGFR of <60 and ≥60 mL/min/1.73 m^2^ ^(14)^. However, this report did not describe the relationship between changes in hemoglobin and sBP and the corresponding baseline values. Matsubayashi et al. ^(15)^ demonstrated that hemoglobin levels significantly increased in subjects with anemia (baseline hemoglobin <130 and <120 g/L in men and women, respectively) and those with hemoglobin levels within the normal range, although hemoglobin significantly decreased in subjects with polycythemia (hemoglobin >165 and >160 g/L in men and women, respectively) at 52 weeks after initiating tofogliflozin in 774 patients with type 2 diabetes according to a *post hoc* analysis of two phase 3 studies. The authors concluded that the hemoglobin levels might approach normal levels after tofogliflozin therapy in patients with both polycythemia and anemia. Murashima et al. ^(16)^ reported that patients taking SGLT2 inhibitors had higher hemoglobin levels and a lower incidence of anemia. The authors also demonstrated that the change in hemoglobin after SGLT2 inhibitor therapy was negatively correlated with the baseline value and that an increase in hemoglobin was observed in patients with an eGFR of ≥15 mL/min/1.73 m^2^. Our study excluded subjects with an eGFR of <15 mL/min/1.73 m^2^, and there were significant negative correlations between changes in hematocrit and hemoglobin and the corresponding baseline values in the low-eGFR group.

This study did not clarify the mechanism underlying the increase in hematocrit and hemoglobin levels after tofogliflozin therapy. Ghanim et al. ^(17)^ reported an improvement in iron utilization, such as decreased hepcidin and ferritin levels and increased transferrin levels, in 52 patients with type 2 diabetes treated with dapagliflozin for 12 weeks. Hepcidin is a key regulator of iron metabolism, and its serum concentration increases with the progression of renal damage in patients with chronic kidney disease (CKD) ^(18)^. Three facts indicate that SGLT2 inhibitors may be effective in treating renal anemia, although anemia is resistant to treatment with erythropoiesis-stimulating agents; the hematocrit and hemoglobin levels increased after SGLT2 therapy in patients whose eGFR had already decreased; greater elevations of hemoglobin and hematocrit were observed in patients with lower corresponding baseline values; and SGLT2 inhibitors improve iron utilization.

In this study, changes in sUA levels were significantly associated with baseline values in both the normal- and low-eGFR groups. A secondary analysis of the EMPEROR-Reduced trial demonstrated that elevated sUA was associated with advanced severity of heart failure in 3,676 patients with heart failure and that empagliflozin reduced the incidence of clinically relevant hyperuricemia, such as gout, gouty arthritis, or antigout therapy initiation ^(19)^. In this analysis, the reduction in sUA 4 weeks after empagliflozin therapy was most pronounced in the highest baseline sUA subgroup categorized by sUA tertiles. The sUA-lowering effect of empagliflozin was consistent among individuals with renal impairment.

Most uric acid reabsorption in the proximal renal tubules occurs via urate transporter 1 (URAT1). SGLT2 inhibitors reduce sUA by increasing the urinary excretion of uric acid via the suppression of URAT1 due to the inhibition of sodium-glucose cotransport and an increase in glucose in the renal tubules ^(20)^. Conversely, the suppressive effect of URAT1 associated with urinary glucose excretion may be attenuated in patients without hyperuricemia because urinary uric acid excretion has already been accelerated. Furthermore, sUA is generally positively correlated with body mass index (BMI) ^(21)^. A positive correlation between sUA and BMI was also found in this study (r_s_ = 0.18, P = 0.06). Because improvement in insulin resistance with tofogliflozin therapy may be more prominent in patients with high BMI, the correction of hyperinsulinemia is considered more pronounced in such patients. Hyperinsulinemia caused by insulin resistance stimulates uric acid reabsorption by regulating URAT1 ^(20), (22)^. Therefore, uric acid reabsorption may be strongly inhibited by tofogliflozin in individuals with insulin resistance (i.e., higher sUA values). These hypotheses could explain the relationships between changes in sUA levels and their corresponding baseline values in the normal- and low-eGFR groups.

Yanai et al. ^(23)^ investigated the changes in metabolic parameters in 229 patients with type 2 diabetes treated with SGLT2 inhibitors, including 26 patients who received tofogliflozin. They reported that changes in blood pressure and sUA at both 3 and 6 months after the start of SGLT2 inhibitor treatment were significantly negatively correlated with the corresponding baseline values. In this study, sBP tended to be positively correlated with BMI (r_s_ = 0.15, P = 0.09). If an increase in urinary sodium excretion secondary to sodium-glucose cotransport inhibition was greater in patients with a high BMI, as mentioned earlier, then the change in sBP was significantly correlated with the baseline value.

All changes in hematocrit, hemoglobin, sBP, uPE, and sUA examined in this study are considered important for the renal prognosis of patients with type 2 diabetes and CKD. Increases in hematocrit and hemoglobin levels were greater in patients with lower corresponding baseline values than in those with higher corresponding baseline values in the low-eGFR group. Furthermore, the correlation between the change in hemoglobin level and the baseline value tended to be stronger in the low-eGFR group than in the normal-eGFR group. Decreases in sBP, uPE, and sUA were also greater in patients with higher corresponding baseline values than in those with higher corresponding baseline values in both the normal- and low-eGFR groups. The combination of these clinical benefits partly contributed to the preservation of eGFR with SGLT2 inhibitor therapy, even in patients with renal impairment. There is a concern that the effects of tofogliflozin may be inferior to those of other SGLT2 inhibitors because tofogliflozin has the shortest half-life among SGLT2 inhibitors ^(24)^. However, it is considered valuable that eGFR was sufficiently preserved with improvement in renal prognosis-related factors in patients with poorer corresponding baseline values after tofogliflozin therapy in patients with type 2 diabetes, although renal impairment was present.

This study has several limitations. First, patients in this study were treated with tofogliflozin, and it is unclear whether the results can be applied to all SGLT2 inhibitors. The currently available SGLT2 inhibitors differ in their selectivity for SGLT2. Although tofogliflozin is highly selective for SGLT2, similar to empagliflozin and dapagliflozin, its selectivity for SGLT2 is smaller ^(25)^. It is currently unclear whether selectivity contributes to the different renal benefits. Second, this study included a relatively small number of patients. Furthermore, blood pressure was measured once during the hospital visit, and the urinary protein level was not a quantitative value corrected for creatinine. Therefore, it is necessary to consider the possibility that the relationship between changes in clinical parameters and their corresponding baseline values occurred incidentally. An analysis of a larger cohort is required to confirm our results. Third, the observation period after initiating tofogliflozin was insufficient. Although a longer observation period is desirable, accurate evaluation of the effects of tofogliflozin is difficult because medications other than SGLT2 inhibitors often change in clinical practice. Patients with severe hypertension, hyperuricemia, or anemia at baseline were excluded from this study because other drug interventions were necessary after initiating tofogliflozin. Fourth, our results demonstrated the relationship between changes in the prognostic factors of CKD and their corresponding baseline values observed in patients with type 2 diabetes, regardless of the presence of renal impairment. Currently, many unknown factors are involved in the mechanisms underlying these relationships.

Despite these limitations, we believe that the results of this study are useful when considering the renoprotective effect of tofogliflozin in patients with type 2 diabetes and renal impairment.

In conclusion, renal prognosis-related factors after initiating tofogliflozin therapy (e.g., hematocrit, hemoglobin, sBP, uPE, and sUA) improved in individuals with poorer baseline values and type 2 diabetes, although renal impairment was present.

## Article Information

### Conflicts of Interest

Suzuko Matsumoto received lecture fees from Eli Lilly Japan KK, Novo Nordisk Pharma Ltd., Astellas Pharma, Kyowa Kirin Co., Ltd., and AstraZeneca KK. Hiroyuki Ito received lecture fees from Novo Nordisk Pharma Ltd., Eli Lilly Japan KK, Sanofi KK, Astellas Pharma, Kowa Company, Ltd., Taisho Pharmaceutical Co., Ltd., Sumitomo Pharma Co., Ltd., Boehringer Ingelheim, Daiichi Sankyo Company, Novartis Pharma KK, Takeda Pharmaceutical Company Ltd., MSD KK, Terumo Corporation, Mochida Pharmaceuticals, Teijin Pharma, Kissei Pharmaceuticals, Mitsubishi Tanabe Pharma Corporation, Sanwa Kagaku Kenkyusho, AstraZeneca KK, Kyowa Kirin Co. Ltd., Otsuka Pharmaceutical Co., Ltd., Bayer Yakuhin, Ltd., EA Pharma Co., Ltd., Ono Pharmaceutical Co., Ltd., and Viatris Inc., and received consulting fees from Becton, Dickinson and Company. Hideyuki Inoue received lecture fees from Novartis Pharma KK, AstraZeneca KK, and Mochida Pharmaceuticals. Shinichi Antoku received lecture fees from Kyowa Kirin Co. Ltd., Sanofi KK, Taisho Pharmaceutical Co., Ltd., Daiichi Sankyo Company, Novo Nordisk Pharma Ltd., Novartis Pharma KK, AstraZeneca KK, and Otsuka Pharmaceutical Co., Ltd. Toshiko Mori received lecture fees from Novartis Pharma KK. Chiaki I, Shun Miura, Tomoko Yamasaki, and Michiko Togane have no conflicts of interest.

### Acknowledgement

The authors thank Tomoko Koyanagi and Sari Shimizu in the secretarial section of Edogawa Hospital for their valuable help with data collection.

### Author Contributions

Suzuko Matsumoto contributed to conception, design, data collection, review of drafts, editing, interpretation, and final approval. Hiroyuki Ito contributed to the conception, design, data collection, analysis, interpretation, writing of the first draft, editing, and final approval. Hideyuki Inoue contributed to data collection, interpretation, and final approval. Chiaki I, Shun Miura, Sinichi Antoku, Tomoko Yamasaki, Toshiko Mori, and Michiko Togane contributed to data collection and final approval.

### ORCiD iD

Hiroyuki Ito: 0000-0002-8143-0082

### Approval by Institutional Review Board (IRB)

This study was conducted in accordance with the principles of the 2013 Declaration of Helsinki. The Ethics Committee of Edogawa Hospital approved the study protocol and waived the need for written informed consent given that the data were analyzed anonymously for this retrospective analysis based on information stored in the hospital (approval number: 2022-14, approval date: May 30, 2022). The trial was registered in the UMIN-CTR, identifier UMIN000048277.

### Data Sharing

The datasets generated during this study are available from the corresponding author upon reasonable request.

## Supplement

Supplementary Appendices Supplementary Appendix 1. Changes in renal prognosis-related factors from the baseline to 12 months after initiating tofogliflozin
Supplementary Appendix 2. Relationships between the changes in HbA1c and estimated glomerular filtration rate (eGFR) and the corresponding baseline values

## References

[1] Heerspink HJL, Stefánsson BV, Correa-Rotter R, et al. Dapagliflozin in patients with chronic kidney disease. N Engl J Med. 2020;383(15):1436-46. 32970396 10.1056/NEJMoa2024816

[2] The EMPA-KIDNEY Collaborative Group; Herrington WG, Staplin N, Wanner C, et al. Empagliflozin in patients with chronic kidney disease. N Engl J Med. 2023;388(2):117-27. 36331190 10.1056/NEJMoa2204233 PMC7614055

[3] Palmer BF, Clegg DJ. Kidney-protective effects of SGLT2 inhibitors. Clin J Am Soc Nephrol. 2023;18(2):279-89. 36220189 10.2215/CJN.09380822 PMC10103214

[4] Ito H, Matsumoto S, Izutsu T, et al. Different renoprotective effects of luseogliflozin depend on the renal function at the baseline in patients with type 2 diabetes: a retrospective study during 12 months before and after initiation. PLoS One. 2021;16(3):e0248577. 33720983 10.1371/journal.pone.0248577 PMC7959360

[5] Ito H, Inoue H, Izutsu T, et al. Changes in the estimated glomerular filtration rate and predictors of the renal prognosis in Japanese patients with type 2 diabetes: a retrospective study during the 12 months after the initiation of tofogliflozin. PLoS One. 2023;18(9):e0292014. 37733761 10.1371/journal.pone.0292014 PMC10513294

[6] Wanner C, Nangaku M, Kraus BJ, et al. How do SGLT2 inhibitors protect the kidney? A mediation analysis of the EMPA-REG OUTCOME trial. Nephrol Dial Transplant. Forthcoming 2024. 10.1093/ndt/gfae032 PMC11361804 38323492

[7] Zaman Z, Roggeman S, Cappelletti P, et al. Evaluation of Aution Max AX-4280 automated urine test-strip analyser. Clin Chem Lab Med. 2001;39(7):649-57. 11522115 10.1515/CCLM.2001.106

[8] Ito H, Matsumoto S, Inoue H, et al. Anemia combined with albuminuria increases the risk of cardiovascular and renal events, regardless of a reduced glomerular filtration rate, in patients with type 2 diabetes: a prospective observational study. Diabetol Int. 2023;14(4):344-55. 37781474 10.1007/s13340-023-00637-x PMC10533775

[9] Ito H, Takeuchi Y, Ishida H, et al. Mild anemia is frequent and associated with micro- and macroangiopathies in patients with type 2 diabetes mellitus. J Diabetes Investig. 2010;1(6):273-8. 10.1111/j.2040-1124.2010.00060.x PMC4014891 24843443

[10] Ito H, Abe M, Mifune M, et al. Hyperuricemia is independently associated with coronary heart disease and renal dysfunction in patients with type 2 diabetes mellitus. PLoS One. 2011;6(11):e27817. 22125626 10.1371/journal.pone.0027817 PMC3220675

[11] Ito H, Antoku S, Abe M, et al. Comparison of the renoprotective effect of febuxostat for the treatment of hyperuricemia between patients with and without type 2 diabetes mellitus: a retrospective observational study. Intern Med. 2016;55(22):3247-56. 27853065 10.2169/internalmedicine.55.6791 PMC5173490

[12] Li J, Neal B, Perkovic V, et al. Mediators of the effects of canagliflozin on kidney protection in patients with type 2 diabetes. Kidney Int. 2020;98(3):769-77. 32470492 10.1016/j.kint.2020.04.051

[13] Doi Y, Hamano T, Yamaguchi S, et al. Mediators between canagliflozin and renoprotection vary depending on patient characteristics: insights from the CREDENCE trial. Diabetes Obes Metab. 2023;25(10):2944-53. 37385955 10.1111/dom.15191

[14] Wanner C, Lachin JM, Inzucchi SE, et al. Empagliflozin and clinical outcomes in patients with type 2 diabetes mellitus, established cardiovascular disease, and chronic kidney disease. Circulation. 2018;137(2):119-29. 28904068 10.1161/CIRCULATIONAHA.117.028268

[15] Matsubayashi Y, Yoshida A, Suganami H, et al. Predictors of haemoglobin levels and of changes in these levels, focusing on anaemia and polycythaemia after administration of the sodium-glucose cotransporter-2 inhibitor tofogliflozin. Diabetes Obes Metab. 2022;24(12):2469-73. 35979908 10.1111/dom.14836 PMC9825934

[16] Murashima M, Tanaka T, Kasugai T, et al. Sodium-glucose cotransporter 2 inhibitors and anemia among diabetes patients in real clinical practice. J Diabetes Investig. 2022;13(4):638-46. 10.1111/jdi.13717 PMC9017638 34797947

[17] Ghanim H, Abuaysheh S, Hejna J, et al. Dapagliflozin suppresses hepcidin and increases erythropoiesis. J Clin Endocrinol Metab. 2020;105(4):e1056-63. 10.1210/clinem/dgaa057 32044999

[18] Troutt JS, Butterfield AM, Konrad RJ. Hepcidin-25 concentrations are markedly increased in patients with chronic kidney disease and are inversely correlated with estimated glomerular filtration rates. J Clin Lab Anal. 2013;27(6):504-10. 24218134 10.1002/jcla.21634 PMC6807340

[19] Doehner W, Anker SD, Butler J, et al. Uric acid and sodium-glucose cotransporter-2 inhibition with empagliflozin in heart failure with reduced ejection fraction: the EMPEROR-reduced trial. Eur Heart J. 2022;43(36):3435-46. 35788657 10.1093/eurheartj/ehac320 PMC9492270

[20] Chino Y, Samukawa Y, Sakai S, et al. SGLT2 inhibitor lowers serum uric acid through alteration of uric acid transport activity in renal tubule by increased glycosuria. Biopharm Drug Dispos. 2014;35(7):391-404. 25044127 10.1002/bdd.1909 PMC4223977

[21] Wang H, Yao J, Ding N, et al. Correlation of uric acid with body mass index based on NHANES 2013-2018 data: a cross-sectional study. Medicine. 2022;101(39):e30646. 36181053 10.1097/MD.0000000000030646 PMC9524866

[22] Toyoki D, Shibata S, Kuribayashi-Okuma E, et al. Insulin stimulates uric acid reabsorption via regulating urate transporter 1 and ATP-binding cassette subfamily G member 2. Am J Physiol Renal Physiol. 2017;313(3):F826-34. 28679589 10.1152/ajprenal.00012.2017

[23] Yanai H, Hakoshima M, Adachi H, et al. Effects of six kinds of sodium-glucose cotransporter 2 inhibitors on metabolic parameters, and summarized effect and its correlations with baseline data. J Clin Med Res. 2017;9(7):605-12. 28611861 10.14740/jocmr3046w PMC5458658

[24] Kasahara-Ito N, Fukase H, Ogama Y, et al. Pharmacokinetics and pharmacodynamics of tofogliflozin (a selective SGLT2 inhibitor) in healthy male subjects. Drug Res. 2017;67(6):349-57. 10.1055/s-0043-104779 28427104

[25] Abdul-Ghani MA, DeFronzo RA, Norton L. Novel hypothesis to explain why SGLT2 inhibitors inhibit only 30%-50% of filtered glucose load in humans. Diabetes. 2013;62(10):3324-8. 24065789 10.2337/db13-0604 PMC3781482

