# Supplementary material for: Effect of Baseline Values of Renal Prognosis-related Factors on Their Changes after Initiating Tofogliflozin Treatment: A Retrospective Study in Japanese Patients with Type 2 Diabetes and Renal Impairment
Source: JMA J. 2024 Sep 6;7(4):571–9. doi: 10.31662/jmaj.2024-0128 (PMC11543293; doi:10.31662/jmaj.2024-0128)

Supplementary Appendix 1. Changes in the factors related to the renal prognosis from the baseline to 12 months after the initiation of tofogliflozin

	All subjects	Groups divided by eGFR at baseline<br>Normal-eGFR	Low-eGFR	P
Hematocrit (%)	1.7±3.0**	2.0±3.1**	1.1±2.8**	0.24
Hemoglobin (g/L)	4±9**	4±10**	2±9*	0.36
sBP (mmHg)	-5±14**	-4±13*	-7±16*	0.39
dBP (mmHg)	-1±11	0±11	2±9	0.32
uPE (mg/dL)	-23.6±58.7**	-14.1±35.3**	-42.9±86.5**	0.12
sUA (μmol/L)	-18±50**	-21±49**	-13±54	0.65
Serum potassium (mEq/L)	0.0±0.3	0.0±0.3	0.0±0.4	0.74
HbA1c (%)	-0.9±1.3**	-1.0±1.3**	-0.7±1.1**	0.06
eGFR (mL/min/1.73 m^2)	-1.2±8.2	-1.9±9.0	0.2±6.0	0.44

* P <0.05, ** P <0.01 vs. corresponding value at the baseline (see Table 1).

sBP, systolic blood pressure; dBP, diastolic blood pressure; uPE, urinary protein excretion; sUA, serum uric acid; eGFR, estimated glomerular filtration rate.

Supplementary Appendix 2. Relationships between the changes in HbA1c and eGFR and the corresponding baseline values

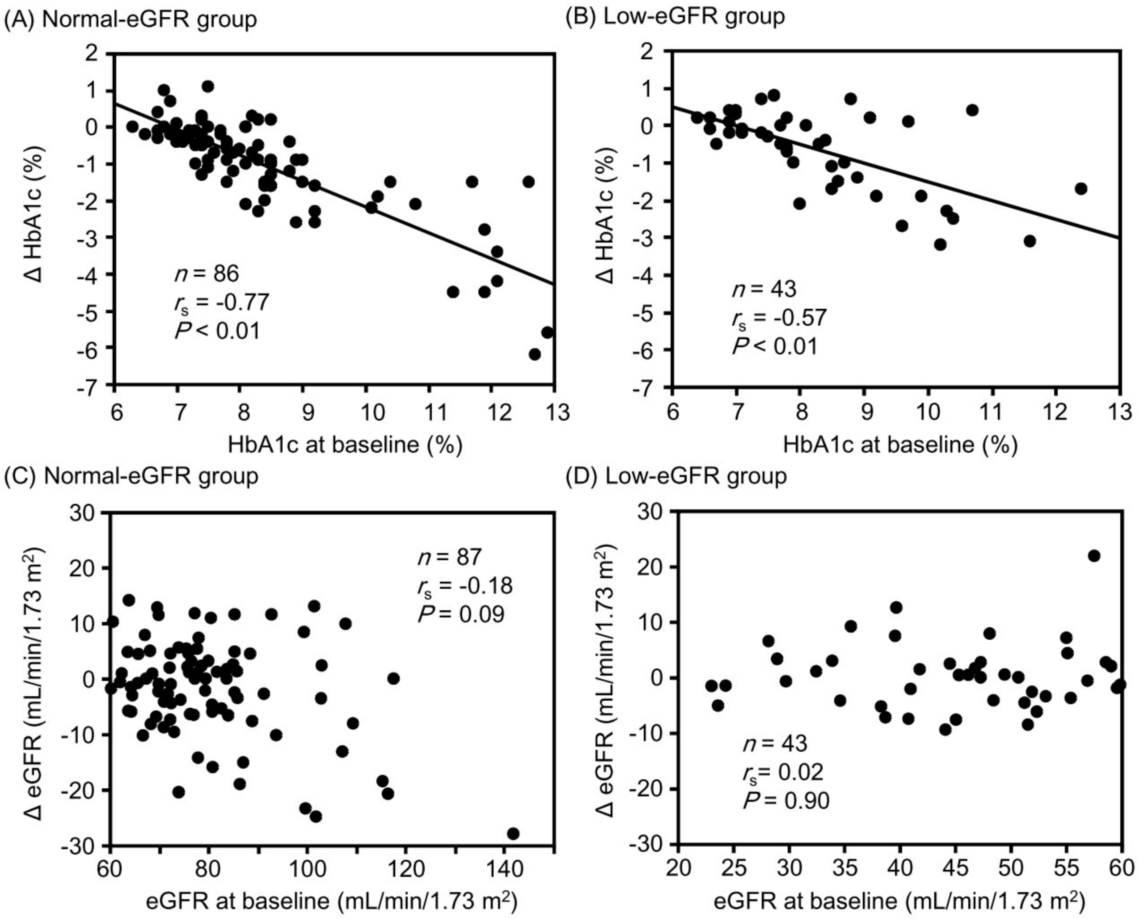

Supplement: Supplementary Appendices — Supplementary Appendix 1. Changes in renal prognosis-related factors from the baseline to 12 months after initiating tofogliflozin Supplementary Appendix 2. Relationships between the changes in HbA1c and estimated glomerular filtration rate (eGFR) and the corresponding baseline values [file 2433-3298-7-4-0571-s001.pdf]
